# Supplementary material for: Impaired adaptation of learning to contingency volatility in internalizing psychopathology
Source: eLife. 2020 Dec 22;9:e61387. doi: 10.7554/eLife.61387 (PMC7755392; doi:10.7554/eLife.61387)
Supplement: Transparent reporting form [file elife-61387-transrepform.pdf]

Westbrook Centre, Milton Road
Cambridge CB4 1YG
UK

P 01223 855340
W elifesciences.org
T @elife

eLife's transparent reporting form

We encourage authors to provide detailed information *within their submission* to facilitate the interpretation and replication of experiments. Authors can upload supporting documentation to indicate the use of appropriate reporting guidelines for health-related research (see EQUATOR Network), life science research (see the BioSharing Information Resource), or the ARRIVE guidelines for reporting work involving animal research. Where applicable, authors should refer to any relevant reporting standards documents in this form.

If you have any questions, please consult our Journal Policies and/or contact us: editorial@elifesciences.org.

Sample-size estimation

- You should state whether an appropriate sample size was computed when the study was being designed
- You should state the statistical method of sample size computation and any required assumptions
- If no explicit power analysis was used, you should describe how you decided what sample (replicate) size (number) to use

Please outline where this information can be found within the submission (e.g., sections or figure legends), or explain why this information doesn't apply to your submission:

Our sample size was informed using previous work (Browning et al., 2015), which observed a medium-sized effect (r=0.4) using a sample size of 30 participants. We estimated that a sample of 75 would be required for 95% power to detect the same effect size. This guided our sample size for experiment 1 (see lines 807-810, page 40).
For our second experiment, we roughly doubled this sample size to allow for potentially poorer data quality when collected online.
Participant numbers can be found on pages 39-40, lines 788-810, for experiment 1 and on page 63, lines 1288-1292, for experiment 2.

Replicates

- You should report how often each experiment was performed
- You should include a definition of biological versus technical replication
- The data obtained should be provided and sufficient information should be provided to indicate the number of independent biological and/or technical replicates
- If you encountered any outliers, you should describe how these were handled
- Criteria for exclusion/inclusion of data should be clearly stated
- High-throughput sequence data should be uploaded before submission, with a private link for reviewers provided (these are available from both GEO and ArrayExpress)

Please outline where this information can be found within the submission (e.g., sections or figure legends), or explain why this information doesn't apply to your submission:

Experiment 2 was a conceptual replication of Experiment 1. It used the reward version of the volatility task from experiment 1, minorly adapted for administration online. For the punishment version of the volatility task, reward loss was used as a replacement for electrical stimulation.
For experiment 1, data from the reward gain or the aversive version of the task were excluded if there was equipment malfunction or if a participant reported after the session that they did not understand the task. Exclusion numbers can be found on pages 39-40, lines 799-807.
For experiment 2, a dataset for a participant was excluded if he/she had greater than ten missed responses in both tasks. Exclusion numbers can be found on page 63, lines 1288-92.

Statistical reporting

- Statistical analysis methods should be described and justified
- Raw data should be presented in figures whenever informative to do so (typically when N per group is less than 10)
- For each experiment, you should identify the statistical tests used, exact values of N, definitions of center, methods of multiple test correction, and dispersion and precision measures (e.g., mean, median, SD, SEM, confidence intervals; and, for the major substantive results, a measure of effect size (e.g., Pearson's r, Cohen's d)
- Report exact p-values wherever possible alongside the summary statistics and 95% confidence intervals. These should be reported for all key questions and not only when the p-value is less than 0.05.

Please outline where this information can be found within the submission (e.g., sections or figure legends), or explain why this information doesn't apply to your submission:

Justifications for data analyses are summarized on page 9 for the bifactor analysis (more details are provided on pages 42-43) and on pages 13-14 for the hierarchical Bayesian modeling of task behavior (more details, pages 45-61).
There were no situations where the N per group was less than 10, so summary statistics are presented (as described below) as opposed to raw data.
For each experiment, we report the posterior mean, as well as the 95% highest density posterior intervals (95%-HDI's), for the cross-group effects (i.e. across all participants) for each learning rate component. For experiment 1, these statistics are presented in Figure 3 and on lines 382-398. For experiment 2, these are presented in Figure 5 – figure supplement 1 and on lines 552-557.
We also present the posterior mean and 95%-HDIs for the effects of individual differences in internalizing factor scores on each learning rate component. For experiment 1, these shown in Figure 4, Figure 4 – figure supplement 1, and on pages 21-25. For experiment 2, these are shown in Figure 5, Figure 5 – figure supplement 2, and on pages 28-31.

(For large datasets, or papers with a very large number of statistical tests, you may upload a single table file with tests, Ns, etc., with reference to sections in the manuscript.)

Group allocation

- Indicate how samples were allocated into experimental groups (in the case of clinical studies, please specify allocation to treatment method); if randomization was used, please also state if restricted randomization was applied
- Indicate if masking was used during group allocation, data collection and/or data analysis

Please outline where this information can be found within the submission (e.g., sections or figure legends), or explain why this information doesn't apply to your submission:

All participants completed all experimental conditions. The order of task blocks by task version was counterbalanced across participants such that roughly an equal number of participants received each of the four possible task-block orderings (page 45 lines 919-24).

Additional data files ("source data")

- We encourage you to upload relevant additional data files, such as numerical data that are represented as a graph in a figure, or as a summary table
- Where provided, these should be in the most useful format, and they can be uploaded as "Source data" files linked to a main figure or table
- Include model definition files including the full list of parameters used
- Include code used for data analysis (e.g., R, MatLab)
- Avoid stating that data files are "available upon request"

Please indicate the figures or tables for which source data files have been provided:

The data and code used to create the figures and fit the hierarchical Bayesian models to the data will be uploaded to a Github repository associated with the first author and this repository will also be shared on the Open Science Framework upon publication of the manuscript.

eLife Sciences Publications, Ltd is a limited liability non-profit non-stock corporation incorporated in the State of Delaware, USA, with company number 5030732, and is registered in the UK with company number FC030576 and branch number BR015634 at the address Westbrook Centre, Milton Road Cambridge CB4 1YG, UK | March 2019
